# Transactions of the Pathological Society

**Published:** 1842-11-12

**Authors:** 


### MEDICAL EXAMINER.

NEW SERIES.

No. 46.] PHILADELPHIA, NOVEMBER 12, 1842. [Vol. I

TRANSACTIONS OF THE PATHOLOGICAL SOCIETY.

*Monday, November 7th.*

The Vice President, Dr. Randolph, in the Chair.

Dr. Pepper read the report of a case of

*Medullary Sarcoma of the Liver perforating the Peritoneum.*

Francis B——, a seaman, of intemperate habits, aged about 50 years, entered the Pennsylvania Hospital June 14th, 1842.

He stated that five years ago, whilst at sea, he had received a severe contusion on the right side; and from that time he has complained of more or less pain in the region of the liver, increased by exposure to cold or sudden atmospheric changes.

*Present condition.*—Pulse 100 per minute, and moderately full; tongue coated brown; skin warm, and of a dark yellow colour; bowels constipated; urine scanty, and of a saffron tint; intelligence perfect, and he states that the yellowness of his skin commenced only two weeks before his admittance into the Hospital. He complains of pain in the epigastric region, which is greatly increased by the slightest pressure, or least motion of his body; he also has occasional vomiting of glairy mucus, with constant nausea. No distinct tumour can be felt below the margin of the ribs; but the right side of the thorax, at its lower part, is more prominent than the left; and percussion is perfectly flat from the margin of the ribs up to the axilla: over the same extent the respiration is entirely absent.

A mercurial purge, administered soon after his entrance, caused copious discharges of clay-coloured fæces.

The following day, June 15th, he used Pil. Hydrarg. gr. ij. three times a day; six cups were applied to the right side, and his diet consisted of gruel, or weak broth.

On the 16th the patient had less vomiting, and felt in all respects more comfortable; but the yellowness of the skin and conjunctiva had slightly increased.

Ung. Tart. Emet. was applied over seat of pain; bowels opened by enema.

There was no change in the symptoms up to the 18th, when, about 6 P. M., he complained of sudden and violent pain in the abdomen, followed by a small, rapid pulse, singultus, foaming at the mouth, and cold extremities. Two hours after, the abdomen was greatly distended and tympanitic, and the superficial veins were enormously congested.

Pulv. Opii. gr. ij., to be followed by a terebinthinate injection, and sinapisms to the extremities.

During the night the hot bath, Carb. Ammoniæ, and milk punch were occasionally used.

19th. Pulse 140, and feeble; features contracted; skin cold; distension of abdomen increased, and respiration very laborious.

By means of a stomach-tube, introduced up the rectum, a large quantity of gas was disengaged, without, however, any apparent diminution in the size of the abdomen.

He expired the same evening, at 6 P. M., just twenty-four hours from the commencement of the severe symptoms.

*Autopsy twenty hours after Death.*

Emaciation but moderate. The cavity of the peritoneum contained about half a gallon of bloody serum: a large clot of blood, weighing about three pounds, was situated behind the stomach and colon, and adhering to a fistulous opening in the under surface of the right lobe of the liver. The opening, about half an inch in diameter, was completely surrounded by a soft cancerous mass, (medullary sarcoma,) and could be distinctly traced into the right branch of the Sinus venæ Portarum. Several tumours, of different dimensions, were disseminated throughout the liver, either imbedded in its substance, or projecting from the surface, and giving to that organ a bosselated appearance. The lobulus quadratus was much enlarged, and infiltrated with soft cancerous matter; and the tumour, thus formed, obstructed the flow of bile by pressing on the neck of the gall-bladder and commencement of the hepatic duct.

The tumours presented a mottled appearance, and were composed of red, yellow, and white, medullary-like matter; a similar substance obstructed the different branches of the vena porta, in its distribution through the liver. Liver much enlarged, and of a light yellow colour. Gall-bladder contracted, and containing about ℥ij. of viscid yellow mucus.

The stomach, near its pyloric extremity, adhered to the liver, and corresponding to the point of adhesion, its inner surface presented an ulcer about the size of a dollar, covered with fungoid granulations, and extending down to the peritoneum; for several inches around the coats of the stomach were much thickened and indurated.

The mesenteric veins were remarkably devoid of blood. The mucous membrane of the bladder and bronchia, and the lining membrane of the heart and aorta, had a decided yellow tint; but no traces of bile could be seen in the stomach or intestines.

The lower lobe of right lung was compressed, and partially indurated by the upward projection of the liver.

The symptoms of cancer of the liver are, in many instances, extremely obscure; one or more tumours projecting below the margin of the ribs, when attended by the cancerous cachexia, render the existence of the disease highly probable; but, unfortunately, these symptoms are not unfrequently absent, as was the case in the present instance. The pain complained of by the patient, for the last few years, may be fairly attributed to the cancerous disease; but it is also true, that the disease not unfrequently runs on to a fatal termination, unattended with pain or even jaundice.

The yellowness of the skin, in the above case, did not supervene until three weeks before death, and was most probably caused by the rapid encroachment of the cancerous mass upon the neck of the gall-bladder, and the commencement of the hepatic duct: several cases of a precisely similar character are reported by Andral, in his Clinique Medicale.

The most interesting point in the present case, is the ulcerated opening between the vena porta and the cavity of the peritoneum: the resulting hæmorrhage fully explains the exsanguine appearance of the mesenteric veins, notwithstanding the obstruction to the portal circulation by the soft cancerous matter; the copious internal hæmorrhage also explains the rapid exhaustion of the vital forces, and the absence of peritoneal inflammation.

---

Dr. Pepper also read the report of a case of

*Cancer of the Stomach and Pancreas.*

Michael D——, aged about 50 years, entered the Pennsylvania Hospital June 30th, 1842.

The patient stated that he had been sick for five months, and that his physician considered his disease to be an affection of the kidneys: the history of his previous symptoms was however very imperfect, and could not be relied upon. The day after admission, he presented the following symptoms. Pulse frequent and feeble; tongue red and smooth; no appetite, and occasional vomiting of a greenish fluid; urine scanty and high coloured; insomnia, with frequent moaning; complains of pain in the lumbar region, and tenderness of the abdomen on the slightest pressure. He is much emaciated, and has the peculiar straw-coloured tint which attends the cancerous cachexia. An immoveable tumour, apparently two inches in diameter, could be distinctly felt behind the umbilicus.

The long duration of the disease, and its evidently malignant character, rendered it highly probable that but little benefit could be derived from any course of treatment: small doses of morphia were administered with the view of relieving pain, and allaying the irritability of the stomach—bowels opened by laxative enemata—diet restricted to gruel.

The following day, July 2d, Dr. Stewardson took charge of the patient, and he was not again seen by me until July 30th; at which time he presented the following symptoms. Bowels obstinately constipated; frequent vomiting of a brown and very offensive fluid; tumour behind umbilicus evidently increased; emaciation extreme, and yellow tint of skin more marked.

Notwithstanding the frequent use of laxative and terebinthinate injections, his bowels could not be moved; and the irritability of the stomach was such, that neither medicine, nor nourishment in any form could be retained.

Died August 3d, thirty-five days after admission into the Hospital.

When the stomach and intestines were elevated, a large tumour was brought into view; commencing near the spleen, it extended across the spine to the concavity of the duodenum, and was evidently formed by the pancreas in a state of scirrhous degeneration. The disease was not, however, confined to this gland, but extended to the adjacent parts; the crura of the diaphragm, and the commencement of the psoas muscle on either side, but more particularly the left, were converted into a gray indurated mass; and the left portion of the root of the mesentery also formed a part of the scirrhous tumour.

The abdominal aorta, ascending cava, and the mesenteric vessels and nerves were surrounded by the same indurated tissue; the vertebra appeared perfectly natural; several convolutions of the small intestine adhered to the diseased mass, and were in part obliterated.

The absorbent glands of the lesser omentum were enormously enlarged and indurated, several of them being at least two inches in diameter.

The anterior face of the stomach, extending from the lesser to the greater omentum, was from one-fourth to half an inch thick, and much indurated; and, excepting the sub-peritoneal cellular tissue, the different coats of the stomach were converted into a white homogeneous mass. The mucous membrane was ulcerated for about three inches in extent, and covered with fungoid granulations; near the pyloric extremity, and extending into the duodenum, this membrane was minutely injected and thickened.

Kidneys, and other abdominal viscera healthy. An indurated mass, about an inch in diameter and resembling scirrhus, was situated in the centre of the left lung.

Origin of aorta somewhat dilated, but not perhaps more than is frequently seen in persons of an advanced age.

The diagnosis of scirrhus of the pancreas, is often rendered very obscure by the intimate connection between this gland, and other more important organs; thus, the head of the pancreas when much enlarged, presses upon the duodenum, or obstructs the ductus communis choledochus; causing vomiting, jaundice, and other symptoms which are generally attributed to disease of the stomach or liver. The only positive sign of this disease, is a deep-seated tumour immoveably fixed behind the umbilicus; whereas, the tumour which generally attends scirrhus of the pylorus, is moveable, more superficial, and situated above and to the right of the umbilicus. The two diseases are not unfrequently combined, through the medium of the lymphatics or by direct adhesion, and the above distinction therefore loses much of its importance.

Judging from the morbid appearances in the above case, it is by no means improbable that the disease first commenced in the stomach; and thence, by means of the lymphatics, extended to the pancreas and adjacent parts, producing the pain in the loins and functional disorder of the kidneys, so much complained of by the patient.

It is well known that in cancer of the stomach, the matters vomited are frequently very offensive, and that the constipation is also very obstinate; but in the present instance there was fœcal vomiting, and constipation such as is rarely seen except in cases of mechanical obstruction. Both the above symptoms are fully explained by the adhesion of several of the convolutions of the intestines to the scirrhous mass.

